# New DNA Plasmid Model for Studying DNA Mismatch Repair Response to the G4 Structure

**DOI:** 10.3390/ijms24021061

**Published:** 2023-01-05

**Authors:** Anzhela V. Pavlova, Nina G. Dolinnaya, Maria I. Zvereva, Elena A. Kubareva, Mayya V. Monakhova

**Affiliations:** 1 Department of Chemistry, Lomonosov Moscow State University, Leninskye Gory 1, Moscow 119991, Russia; 2 Belozersky Institute of Physico-Chemical Biology, Lomonosov Moscow State University, Leninskye Gory 1, Moscow 119991, Russia

**Keywords:** G-quadruplex, plasmid DNA, DNA mismatch repair, MutS, MutL, MutH

## Abstract

G-quadruplexes (G4s), the most widely studied alternative DNA structures, are implicated in the regulation of the key cellular processes. In recent years, their involvement in DNA repair machinery has become the subject of intense research. Here, we evaluated the effect of G4 on the prokaryotic DNA mismatch repair (MMR) pathway from two bacterial sources with different mismatch repair mechanisms. The G4 folding, which competes with the maintenance of double-stranded DNA, is known to be controlled by numerous opposing factors. To overcome the kinetic barrier of G4 formation, we stabilized a parallel G4 formed by the d(GGGT)_4_ sequence in a DNA plasmid lacking a fragment complementary to the G4 motif. Unlike commonly used isolated G4 structures, our plasmid with an embedded stable G4 structure contained elements, such as a MutH cleavage site, required to initiate the repair process. G4 formation in the designed construct was confirmed by Taq polymerase stop assay and dimethyl sulfate probing. The G4-carrying plasmid, together with control ones (lacking a looped area or containing unstructured d(GT)_8_ insert instead of the G4 motif), were used as new type models to answer the question of whether G4 formation interferes with DNA cleavage as a basic function of MMR.

## 1. Introduction

Over the past few decades, G-quadruplexes (G4s), a unique class of highly stable four-stranded noncanonical DNA and RNA structures, have been extensively studied in various aspects, from the G4 conformation and biophysical properties to the role of G4s in complex biological processes [1,2]. Endogenous G4s are formed by G-rich nucleic acids whose sequence contains four runs of at least three consecutive guanosines (G-tracts). G-tracts fold intramolecularly to form stacked planar G-tetrads with guanine bases from each tract linked via Hoogsteen hydrogen bonds between adjacent guanines in the tetrad. The quadruplex core is additionally stabilized by the coordination of guanine O6 with monovalent cations (usually K^+^). Nucleotide sequences between G-tracts of G4 can form loops that play an important role in determining the topology and stability of G4s. DNA quadruplexes can have a wide range of folds; some of them are highly dynamic, while others take only one conformation [3]. Under in vitro conditions, DNA G4s adopt parallel, antiparallel, and hybrid (3+1) topologies characterized by different orientations of the four G-tracts. 

Several computational tools such as Quadparcel [4] and G4Hunter [5] have shown that G4-forming sequences (G4 motifs) are often clustered at key genome elements such as telomeric DNA, oncogene promoter regions, mitochondrial DNA, replication origins, genes of ribosomal RNA, etc.

The cellular functions of G4s have been widely investigated, and there is strong evidence that DNA G4s, which interact with numerous cellular proteins and enzymes, are involved in essential genomic processes in the cells of all organisms, including humans: DNA replication and repair [6,7], DNA recombination [8], transcription, telomere maintenance, mutagenesis [1,9,10,11,12,13], and others. However, the true role that G4 structures play in cells, as well as the balance of their favorable and detrimental effects on cellular processes, remains to be determined.

In recent years, the deleterious effect of G4s on genome integrity and their role in the regulation of the DNA repair machinery have become the subject of intense research. In this study, we focused on evaluating the G4-driven response of the DNA mismatch repair (MMR) system, which plays a critical role in maintaining genome stability. A thorough study of G4s’ effect on MMR functions in vitro requires DNA models containing the G4 structure, on the one hand, and some functional elements such as the MutH cleavage site, on the other hand. We recently described the design and preparation of a linear double-stranded DNA with an embedded stable extrahelical G4 and a set of DNA sites required for MMR initiation [14]. It has been shown that despite the high binding affinity of G4 to the major MMR proteins, MutS and MutL, from *Escherichia coli* (ecMutS and ecMutL, respectively), the G4 structure is not recognized by MMR as a repair signal; nevertheless, it does not prevent MMR processing when the G4 and G/T mismatch are in close proximity [7].

Our finding that ecMutS specifically binds to the biologically relevant parallel G4, which is “frozen” in a DNA duplex context, with stronger affinity than to G/T mismatch in the presence of a nonhydrolyzable ATP analog, is consistent with the previously described ecMutS binding to isolated intermolecular DNA G4 [15]. These data support the hypothesis of Ehrat et al. that MutS is unable to activate the ATP-dependent canonical MMR pathway in response to G4 recognition [15]. Using more advanced G4 models that are more similar to those realized in vivo, we found experimental evidence that ecMutS binding modes to mismatched and G4-containing DNAs are different [14].

Although our constructs with a biologically relevant intramolecular G4 stabilized in a duplex surrounding that contains the selected functional moieties (e.g., protein recognition sites, DNA lesions, signaling sequences, etc.) are more useful for studying multicomponent G4-driven biological processes than isolated G4 structures, they carry limitations such as insufficient length of DNA substrates. The latter is important for the formation of a stable complex between DNA and different MMR proteins [16,17]. Moreover, the use of covalently closed plasmid DNA allows the nonspecific interaction between MutS and the DNA ends to be avoided [18], which may affect the MMR activation process.

Here, we designed and prepared a previously unreported plasmid DNA construct as a novel type model for studying the G4-mediated DNA mismatch repair response. Our approach is based on the well-known protocol for incorporating oligonucleotides containing specific DNA lesions into a gapped DNA plasmid [19]. In this method, a nicking endonuclease produces a single-stranded DNA stretch on a DNA plasmid to be replaced by a synthetic oligonucleotide, after which the DNA ligase seals the nick sites. We modified this approach to insert into the DNA plasmid not only a mismatched G/T base pair, but also an intramolecular parallel G4, stabilized in a duplex context due to the lack of a DNA sequence complementary to the G4 motif, and MutH cleavage site required to initiate the *E. coli* MMR pathway. G4 formation in the engineered plasmid construct was confirmed by Taq polymerase stop assay and dimethyl sulfate (DMS) probing. This model was used to evaluate the role of G4 in the initiation of the MMR pathways from two bacterial sources with different mismatch repair mechanisms.

## 2. Results and Discussion

DNA mismatch repair is required for genome maintenance by protecting against noncanonical base pairs and insertion/deletion loops due to errors during DNA replication or homologous recombination. It has been proven that the basic features of MMR have been conserved throughout evolution from bacteria to humans [20]. The most studied and widely employed MMR systems are those of *E. coli* and humans. In *E. coli*, the repair process is initiated by binding of the ecMutS protein to mismatched DNA bases. Upon recognition of the mismatch, ecMutS recruits ecMutL in an ATP-dependent manner to form a ternary complex that is thought to coordinate a cascade of downstream events. ecMutL stimulates the *E. coli* MutH (ecMutH) endonuclease, which interprets the lack of DNA methylation at its recognition site–5′-GATC-3′/3′-Gm^6^ATC-5′ (where m^6^A corresponds to N6-methyl-2′-deoxyadenosine), as a daughter strand mark, thereby helping to distinguish and cleave the newly synthesized strand (methyl-directed MMR). The unmethylated DNA strand is then hydrolyzed by a set of exonucleases. Finally, DNA polymerase and ligase fill in the gap in the daughter strand (for review, see [21]). Eukaryotes and most bacteria lack MutH endonuclease. In these organisms, the MutL itself has endonuclease activity that is independent of daughter strand methylation (methyl-independent MMR). 

### 2.1. Design and Production of Novel MMR Substrates Based on Double-Gapped DNA Plasmids

Obviously, a new type of double-stranded DNA substrates are needed to monitor G4-mediated responses of MMR proteins. They must be sufficiently long, covalently closed (without free ends), and contain not only a stable G4 structure but also DNA sites required for MMR initiation.

With the exception of the single-stranded telomeric ends, all genomic G-rich sequences are always present along with their C-rich complements. The formation of G4, which competes with the preservation of the DNA double helix, is regulated by various oppositely directed cellular factors: nucleosome-free chromatin state; negative supercoiling; biological processes that locally open double-stranded DNA, such as DNA replication, transcription, recombination, and DNA repair; G4-stabilizing cellular proteins; as well as the exclusion of the C-rich strand from the G4–DNA duplex equilibrium due to the formation of a stable i-motif or co-transcriptional R-loop [7]. Several in vitro studies have shown that, under physiological conditions, isolated G4 motifs typically form DNA duplexes in the presence of a complementary C-rich strand rather than G4s [22,23]. We recently created a stable analog of a G4 in the double-helical context by hybridization of partially complementary strands, one of which contained the G4 motif d(GGGT)_4_ flanked by oligonucleotide fragments, while the opposite strand lacked a site complementary to the G4-forming insert [14].

**Figure 1:**
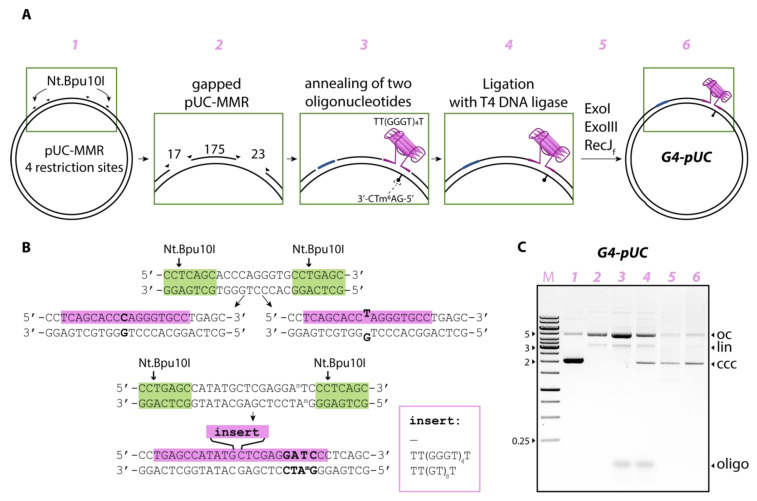
Construction of new type MMR models based on Dcm- and Dam-methylated pUC-MMR plasmid DNA. (**A**) Scheme of Nt.Bpu10I endonuclease-mediated double-gapped plasmid generation allowing replacement of 17- and 23-nt DNA fragments to introduce a stable parallel G4 (a schematic representation of the G4 structure is shown in pink) and monomethylated ecMutH recognition site, 5′-GATC-3′/3′-Gm^6^ATC-5′. (**B**) Overview of the assembly of the target G4-pUC plasmid and control plasmids with ecMutH recognition site and with or without G/T mismatch. Key elements of the plasmid models, including DNA mismatch, G4 structure or loop, and the ecMutH recognition site, are in bold. The Nt.Bpu10I recognition site is highlighted in green, and the oligonucleotide sequences used to replace original pUC-MMR sequences are highlighted in pink. (**C**) Monitoring the steps of G4-pUC creation by electrophoresis in 1% agarose gel prestained with ethidium bromide; the positions of open circle DNA (oc), linearized DNA (lin), covalently closed circular DNA (ccc), and gap-filling oligonucleotides (oligo) are indicated on the right. Marker DNA length (in kb) is shown on the left. The numbers denoting the steps of obtaining modified plasmids (from *1* to *6*) at the top of panels (**A**,**C**) match.

In this study, a stable parallel G4 formed by the same sequence was inserted not into a linear DNA duplex but into plasmid DNA along with the MutH recognition site. To obtain this new type of MMR substrate, we modified and adapted the known protocol for preparing gapped plasmid DNA followed by ligation of oligonucleotides containing certain DNA elements into a gapped molecule [19,24,25]. We used the Nt.Bpu10I endonuclease to introduce four nicks into the same strand of the pUC-MMR plasmid obtained from *dam^+^*, *dcm^+^ E. coli* cells [26], resulting in a double-gapped product lacking 17- and 23-nt oligomers; gaps were separated by 175 base pairs (bp) (Figure 1A). Oligonucleotides 17G/C or 17G/T providing Watson–Crick’s G/C or mismatched G/T base pairs, respectively, were inserted using annealing and ligation procedures into the 17-nt gap (Table 1, Figure 1B). Another gap of 23-nt residues located between the second pair of nicks was used to insert the 42-nt 42G4 oligomer into the same plasmid without mismatch (Table 1, Figure 1B). This oligonucleotide contained the d(TT(GGGT)_4_T) insert capable of folding into a parallel G4 structure, as well as its flanks, complementary to the sequence opposite the 23-nt gap. As a result, intramolecular G4 was stabilized in a double-stranded DNA context because it could not flip into B-DNA and compete with G4 formation due to the lack of a complementary sequence in the engineered plasmid construct. Although any natural or modified DNA plasmids have negative supercoiling, which contribute to the local opening of the DNA double helix, it has been reported that this factor is in some cases insufficient to favor the G4 formation by the G4 motif, which is part of the B-DNA [7]. Generally, additional G4 stabilizing factors are required to overcome the energy barrier to melt an extended stretch of the DNA double helix during G4 folding in vitro and in vivo. For this reason, we excluded C-rich complement from the G4-DNA duplex equilibrium.

It should be noted that in the Dcm- and Dam-methylated pUC-MMR plasmid, adenosine residues in both strands are methylated at the N6 position of A in each 5′-GATC-3′/3′-GATC-5′ site. Oligonucleotide 42G4 containing the unmethylated sequence d(GATC) was used to obtain the monomethylated ecMutH recognition site (see above) (Table 1); it replaced the methylated fragment in the 23-nt gap of the original plasmid. The potential G4 structure turned out to be in close proximity (6 bp) to the monomethylated ecMutH recognition site located in the duplex fragment flanking the G4 motif (Figure 1A). 

The control plasmid systems differed from the G4-containing G4-pUC by the absence of the G4 motif (G/T-pUC or G/C-pUC) or by the replacement of the d(GGGT)_4_ insert by the d(GT)_8_ sequence of the same size (loop-pUC) that is unable to form a stable G4 structure within the looped area [27]. The summary of the combinations of synthetic oligonucleotides filling the double-gapped plasmid molecule to create various target and control MMR substrates containing structural and/or functional elements is presented in Table 2. The assembly process of the modified pUC-MMR plasmids was visualized step-by-step by electrophoresis in 1% agarose gel prestained with ethidium bromide, which help to visualize covalently closed circular (ccc) DNA, which migrates faster during gel electrophoresis, open circle nicked (oc), and linearized (lin) DNA (see, for example, Figure 1C).

### 2.2. Monitoring of the G4 Structure on a Plasmid DNA Construct

**Figure 2:**
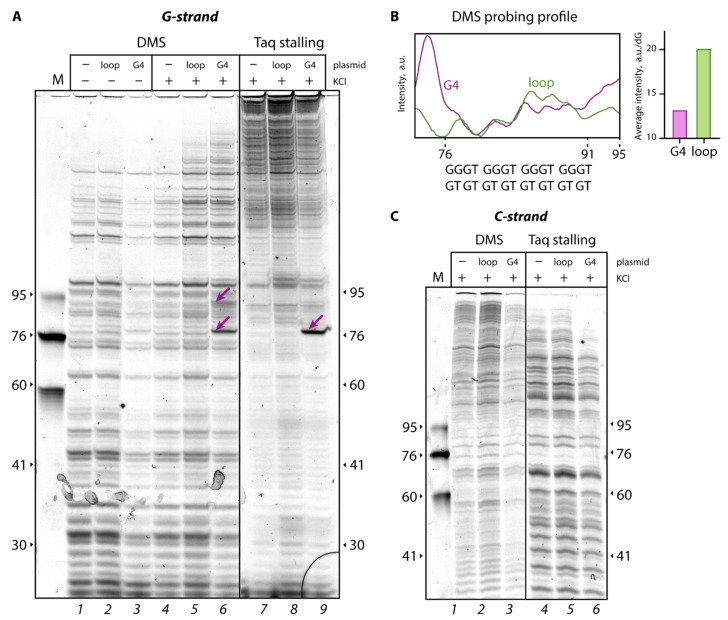
Monitoring of the G4 structure in engineered plasmid DNAs. (**A**) Data from DMS probing followed by Taq polymerase primer extension (lanes *1*–*6*) and Taq polymerase stop assay (lanes *7*–*9*) for the G-strand. TAMRA-labeled primer G (Table 1) was used. Unmodified pUC-MMR plasmid (lanes *1*, *4*, and *7*), loop-pUC plasmid (lanes *2*, *5*, and *8*), and G4-pUC plasmid (lanes *3*, *6*, and *9*), designated as –, loop, and G4, respectively, used in the comparative experiments. The absence or presence of K^+^ in reaction was indicated by “−“ or “+”, respectively. Oligonucleotide products of the Taq polymerase stop assay and DMS probing followed by primer extension reaction were analyzed by 10% polyacrylamide gel electrophoresis under denaturing conditions. The length of the oligonucleotides used as markers is shown to the left and right of the electrophoregrams. The arrows correspond to the high-intensity bands discussed in the text. (**B**) Densitometric profile of fluorescence primer extension products after DMS modification and piperidine solution cleavage for the 76–95-nt region of lanes *5* and *6* in the electrophoregram in the (**A**). Mean band intensities for one of the twelve guanosines in the d(GGGT)_4_ motif (G4-pUC plasmid) and one of the eight guanosines in the d(GT)_8_ sequence (loop-pUC plasmid) are plotted on the right. (**C**) Data from DMS probing followed by primer extension reaction (lanes *1*–*3*) and Taq polymerase stop assay (lanes *4*–*6*) for the partially complementary C-strand: unmodified pUC-MMR plasmid (lanes *1* and *4*), loop-pUC plasmid (lanes *2* and *5*), G4-pUC plasmid (lanes *3* and *6*). TAMRA-labeled primer C (Table 1) was used. The designation for plasmids and other labels are the same as in (**A**).

To confirm the formation of the G4 structure in the G4-pUC plasmid, Taq polymerase stop assay was used as a sensitive and specific tool to detect noncanonical DNA structures, which constitute an obstacle to replication machinery [28]. Extension of the TAMRA-labeled primer G (Table 1) annealed to the sequence by 76 bp upstream of the G4 motif, was paused immediately before the d(GGGT)_4_ sequence, resulting in a clear band corresponding to a ~76-nt product on a denaturing polyacrylamide gel (Figure 2A, lane *9*). An effective primer extension pause site is observed in the presence of 50 mM KCl, which promotes G4 formation. At the same time, the use of the d(GT)_8_ sequence instead of the d(GGGT)_4_ motif did not lead to Taq polymerase arrest (Figure 2A, lane *8*); full-sized products predominated in the reaction mixture.

For the direct detection of the G4 structure incorporated into plasmid constructs, DMS chemical probing followed by primer extension reaction was used as an independent method [29]. This reagent most effectively methylates the N7 position of guanine residues, and the reaction efficiency depends on the DNA secondary structure. DMS modification leads to easy depurination and strand cleavage upon treatment with piperidine water solution. Because N7 of guanines involved in G-tetrads is protected from chemical modification by Hoogsteen pairing, DMS probing has been widely utilized to assess G4 formation and its distinction from double- and single-stranded DNA regions [30]. DMS probing was performed in the presence of 100 mM KCl to facilitate G4 formation and in the absence of K^+^ for control samples (Figure 2A, lanes *4*–*6* and *1*–*3*, respectively). To select the zone of interest in the plasmid constructs, the samples after treatment with DMS and piperidine water solution were subjected to a Taq polymerase primer extension reaction with a fluorescently labeled primer G (Table 1). Reaction products were visualized by polyacrylamide gel electrophoresis under denaturing conditions. As shown in Figure 2B, the G-tracts in the G4-pUC plasmid (the region of ~76–91-nt products in the G-rich strand, referred to as G-strand) manifested a 1.5-fold reduction in fluorescence intensity of primer extension products compared to that of the same region in the loop-pUC plasmid with extrahelical d(GT)_8_. These data confirm the involvement of guanines from the d(GGGT)_4_ insert in Hoogsteen base pairing and indicates the successful G4 formation in the G4-pUC plasmid. Interestingly, intense bands up- and downstream of the G4 motif (Figure 2A, lane *6*) suggest hypersensitivity of the G4–DNA duplex junction to DMS modification, indicating an unpaired state of the G residues adjacent to the G4 structure.

Notably, the band of the 76-nt primer extension product corresponding to the Taq polymerase pause site loses intensity after G4-pUC modification with DMS under conditions unfavorable for G4 formation (in the absence of potassium ions) (Figure 2A, lane *3*).

Meanwhile, analysis of Taq polymerase stalling and DMS modification applied to a partially complementary strand without any inserts (referred to as C-strand) did not show any difference in band patterns between the G4-pUC and loop-pUC plasmids (Figure 2C).

### 2.3. The Role of G4s in the Initiation of the MMR Process in E. coli and Neisseria gonorrhoeae, Manifested by Cleavage of the DNA Substrates

Strong specific interactions of G4s with MMR proteins MutS and MutL from various organisms including *E. coli*, *N. gonorrhoeae*, *R. sphaeroides*, and humans have been described previously [14,15,31,32]. However, the biological significance of G4 recognition by cellular proteins is not always clear. To answer the question of whether G4 formation interferes with basic MMR functions, we examined the G4 effect on cleavage of the unmethylated strand at the monomethylated 5′-Gm^6^ATC-3′/3′-GATC-5′ site using ecMutH coordinated by ecMutS and ecMutL, as well as on the nicking activity of MutL from *N. gonorrhoeae* (ngMutL), which itself has an endonuclease function. The modified DNA plasmids G4-pUC, G/T-pUC, G/C-pUC, and loop-pUC (Table 2) were used as the MMR substrates and reference systems.

**Figure 3:**
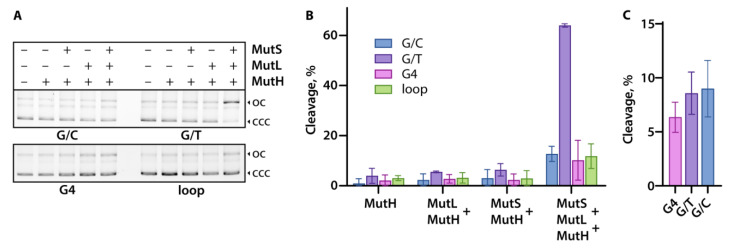
Cleavage of G4-pUC and control pUC plasmids (Table 2) by MMR proteins. (**A**) Cleavage products of the plasmid substrates G/C-pUC, G/T-pUC, G4-pUC, and loop-pUC, referred to as G/C, G/T, G4, and loop (10 nM), by *E. coli* MMR proteins were analyzed by electrophoresis in 1% agarose gel containing ethidium bromide; the positions of open circle (nicked) DNA (oc) and covalently closed circular DNA (ccc) are indicated on the right. The reaction mixtures were incubated in the buffer solution containing 1 mM ATP, 5 mM MgCl_2_, and 120 mM KCl for 1 h at 37 °C in the presence of 100 nM ecMutH alone, 100 nM ecMutH and 250 nM ecMutL and/or 250 nM ecMutS (all protein concentrations are calculated per monomer). (**B**) Efficiency of plasmid hydrolysis induced by ecMutH alone and combinations of ecMutH with ecMutS and/or ecMutL. Reaction conditions are indicated in panel (**A**). (**C**) ngMutL-induced (1 μM per monomer) hydrolysis of plasmid substrates (10 nM). The reaction mixtures were incubated in the presence of 5 mM MnCl_2_, 50 mM KCl for 1 h at 37 °C, and then electrophoresed in 1% agarose gel containing ethidium bromide.

ecMutH- or ngMutL-mediated nicking of plasmid constructs, leading to the formation of a relaxed open circle DNA, was detected by electrophoresis in 1% agarose gel prestained with ethidium bromide (Figure 3). To evaluate the cleavage efficiency, plasmid nicking products were analyzed for the ecMutH alone (100 nM) as well as for ecMutH in the presence of ecMutS, ecMutL, or both proteins at equal concentrations of 250 nM per protein monomer (Figure 3A,B). ecMutH alone or ecMutH coordinated only by one of ecMutS or ecMutL has equally low cleavage efficiency (~2–4%) on all tested plasmid DNAs. At the same time, the entire set of *E. coli* MMR proteins, ecMutH, ecMutS, and ecMutL, was found to be able to discriminate G/T mismatch in the plasmid G/T-pUC without G4 or d(GT)_8_ inserts as a cognate DNA lesion, and initiate the MMR system. Although the cleavage efficiency of the remaining plasmids, including G4-pUC, with the complete set of ecMutH–ecMutL–ecMutS proteins (~10–12%) was 4–5 times higher than with the combination of ecMutH–ecMutL or ecMutH–ecMutS, it was still 5 times lower than for G/T-pUC under the same conditions and similar to that of canonical G/C-pUC. These findings support data obtained for a linear DNA model with intramolecular parallel G4 stabilized in a duplex context [14] and prove that the G4 structure, in contrast to G/T mismatch, does not activate the *E. coli* MMR system.

In addition, we aimed to answer the question of whether a single G4 inserted into a DNA plasmid can affect the activity of ngMutL with an endonuclease function in the methyl-independent MMR inherent in eukaryotic organisms. It has previously been shown that ngMutL recognizes and preferentially binds isolated DNA G4 with high affinity, but, nevertheless, inefficiently cleaves the phosphodiester bonds inside of the G4-motif in *TERT* promoter fragment [32]. To clarify this issue, we analyzed ngMutL-mediated cleavage of the G4-pUC plasmid, which contained an extrahelical stable G4, as well as reference plasmids, G/C-pUC and G/T-pUC. The ngMutL protein was found to nick all plasmid MMR substrates in the presence of Mn^2+^ with the same efficiency (Figure 3C), confirming that the G4 structure does not activate methyl-independent MMR endonuclease MutL.

## 3. Materials and Methods

### 3.1. Oligonucleotides and Plasmid DNA

Oligodeoxyribonucleotides (Table 1), including those carrying a TAMRA fluorescent label at the 5′-end, were synthesized by standard phosphoramidite chemistry and purified using high-pressure liquid chromatography (Evrogen, Moscow, Russia). Oligonucleotide strand concentrations were determined spectrophotometrically; extinction coefficients were derived from nearest-neighbor data (OligoAnalyzerTM Tool. Available online: https://www.idtdna.com/calc/analyzer (accessed on 30 August 2020)).

pUC-MMR plasmid was kindly provided by Prof. P. Friedhoff (Justus Liebig University, Giessen, Germany) and further purified from *dam^+^*, *dcm*^+^ XL1 Blue *E. coli* strain (Stratagene, La Jolla, CA, USA). Plasmids containing the *mutS*, *mutL*, and *mutH* genes from *E. coli* were kind gift from Prof. P. Friedhoff. The plasmid for ngMutL expression was provided by Prof. D.N. Rao (Indian Institute of Science, Bangalore, India).

### 3.2. Enzymes and Proteins

Nt.Bpu10I nicking endonuclease, T4 DNA ligase, exonucleases ExoI and ExoIII, bovine serum albumin (BSA) were purchased from Thermo Fisher Scientific (Waltham, MA, USA); RecJ exonuclease was obtained from New England BioLabs (Ipswich, MA, USA).

Recombinant 6xHis-tagged ecMutS, ecMutL, and ecMutH proteins, as well as 6xHis-tagged ngMutL were isolated from BL21(DE3) *E. coli* strain and purified by Ni-NTA affinity and size-exclusion chromatography on Superdex 200 TM 10/300 (GE Healthcare, Chicago, IL, USA) on Äkta Purifier (GE Healthcare, Chicago, IL, USA) as previously described [33,34].

### 3.3. Construction of Modified DNA Plasmids Used in This Study

Various MMR plasmid substrates (Table 2) were generated using a procedure similar to that described previously [19]. To produce plasmid DNA containing two gapped sites, Dam- and Dcm-methylated pUC-MMR plasmid (110 μg, 50 nM) [26] was incubated with Nt.Bpu10I nicking endonuclease (0.012 units/μL) in 1 mL of 10 mM Tris-HCl buffer (pH 8.5) containing 10 mM MgCl_2_, 100 mM KCl, and 0.1 mg/mL BSA for 3 h at 37 °C. The reaction was terminated by incubating the reaction mixture for 20 min at 65 °C. The nicked plasmid was precipitated with an ethanol–NaOAc solution and resuspended in 1 mL of T4 ligase buffer from Thermo Fisher Scientific (50 mM Tris-HCl (pH 7.5), 10 mM MgCl_2_, 1 mM ATP, 10 mM DTT). To fill gaps in the plasmid and create DNA constructs containing G/T mismatch, control canonical G/C base pair, stable G4 structure, or extrahelical loop, combinations of two chemically synthesized oligonucleotides including 17G/T, 17G/C, 42G4, 42loop, and 23GATC (Table 2) were added to the mixture to a concentration of 9 µM, providing 180-fold excess of each oligonucleotide over the plasmid concentration. The samples were heated for 10 min at 80 °C and slowly cooled to room temperature over several hours. Then, ATP and DTT were added to the reaction mixtures to final concentrations of 1 mM and 10 mM, respectively. The annealed samples were incubated with 0.075 units/µL T4 DNA ligase for 14 h at 37 °C to produce intact modified plasmid DNAs. The ligation products were purified from nicked and linear DNA by incubation with exonucleases ExoI (0.05 units/µL), ExoIII (0.12 units/µL), and RecJ (0.05 units/µL) for 14 h at 37 °C followed by column purification with a NucleoSpin Gel and PCR clean-up kit (Thermo Fisher Scientific, Waltham, MA, USA). The overall yield was 30–40%. Aliquots of plasmid DNA samples at various stages of transformation were analyzed by electrophoresis in 1% agarose gel, prestained with 0.5 μg/mL ethidium bromide, using Tris-acetate-EDTA buffer. Gels were visualized using ChemiDoc XRS+ (Bio-Rad, Hercules, CA, USA).

### 3.4. Taq Polymerase Stop Assay

Plasmids were subjected to a primer extension reaction as described previously [28]. The reaction mixture (10 µL) included ~5 nM plasmid DNA, 0.1 µM oligonucleotide primer G or primer C (Table 1), and 0.6 units of Taq polymerase in 10 mM Tris-HCl buffer (pH 8.8) containing 10 μM mixture of four dNTPs (dATP, dGTP, dCTP, dTTP), 50 mM KCl and 5 mM MgCl_2_. The primer extension reaction included 35 cycles: annealing for 30 s at 90 °C, primer annealing for 1 min at 53 °C, and elongation for 1 min at 72 °C. The reaction mixture was supplied with 10 µL of formamide and analyzed by electrophoresis in a 10% polyacrylamide gel containing 7 M urea. DNA fragments were visualized with a Typhoon FLA 9500 (GE Healthcare, Chicago, IL, USA).

### 3.5. Chemical Probing Assay with DMS Followed by Taq Polymerase Primer Extension

Chemical modification of plasmid DNA samples with DMS followed by primer extension was performed according to the standard procedure [29]. Plasmids (75 µg) were incubated in 50 µL of 20 mM HEPES buffer (pH 7.9) containing 0 or 100 mM KCl in the presence of 1% DMS for 1 min at room temperature. The reaction was terminated by adding 20 µL of 10% 2-mercaptoethanol in 100 mM NaOAc solution. The modified DNA was precipitated with an ethanol–NaOAc solution and cleaved with 10% piperidine water solution at 90 °C for 30 min. After evaporation of the piperidine solution, the cleavage products were dissolved in 30 µL of bidistilled water. Primer extension (38 cycles: annealing for 30 s at 95 °C, primer annealing for 1 min at 53 °C, elongation for 1 min at 72 °C) was performed using 2.5 µg of the reaction product treated with DMS and 10% piperidine solution, 6 units of Taq polymerase in 10 µL of buffer containing 50 mM KCl, 5 mM MgCl_2_, 10 µM mixture of four dNTP (dATP, dGTP, dCTP, dTTP), and 0.2 µM 5′-TAMRA-labeled primer G or primer C (Table 1). Primer extension products were separated by electrophoresis in a 10% polyacrylamide gel containing 7 M urea. DNA fragments were visualized with a Typhoon FLA 9500 (GE Healthcare, Chicago, IL, USA). 

### 3.6. Single-Strand Cleavage of Plasmid DNAs by ecMutH

Plasmid DNAs (10 nM) were incubated with 100 nM ecMutH (with or without 250 nM ecMutS and 250 nM ecMutL (all protein concentrations were calculated per monomer) in 10 µL of 20 mM HEPES-KOH buffer (pH 7.9) containing 120 mM KCl, 5 mM MgCl_2_, 1 mM ATP, and 0.5 mg/mL BSA for 1 h at 37 °C. The reaction was terminated by adding 1 mg/mL proteinase K followed by incubation at 55 °C for 20 min. Samples were analyzed by electrophoresis in 1% agarose gel containing 0.5 μg/mL ethidium bromide using Tris-acetate-EDTA buffer. Gels were visualized with ChemiDoc XRS+ (Bio-Rad, Hercules, CA, USA). Cleavage efficiency was calculated from at least three independent experiments by subtracting the percentage of nicked plasmid in the control sample from the total percentage of target nicked plasmid determined using TotalLab TL120 software (Nonlinear Dynamics Ltd., New Castle, UK). Errors are presented as 95% confidence intervals.

### 3.7. Nicking Endonuclease Activity of ngMutL

Plasmid MMR substrates (10 nM) were incubated in 10 μL of 20 mM HEPES-KOH buffer (pH 8.0) containing 50 mM KCl, 5 mM MnCl_2_, 1 mM DTT, 7% (*v*/*v*) glycerol with 1 μM (per monomer) ngMutL for 1 h at 37 °C. The reaction was terminated by adding 50 mM EDTA and 1 mg/mL proteinase K, followed by incubation at 55 °C for 20 min. The products were electrophoresed in 1% agarose gel in Tris-acetate-EDTA buffer containing 0.5 μg/mL ethidium bromide. Gels were visualized using a ChemiDoc XRS+ (Bio-Rad, Hercules, CA, USA). Cleavage efficiency was calculated from two independent experiments using TotalLab TL120 software (Nonlinear Dynamics Ltd., New Castle, UK). Errors are presented as 95% confidence intervals.

## 4. Conclusions

Recent studies have shown that G4 structures contribute to genomic instability. To clarify whether G4s can interfere with the MMR pathway, we developed a new type of model based on plasmid DNA. This model contains a G4 structure stabilized in a duplex context, along with other elements required for initiation of DNA mismatch repair. The procedure for preparing of G4-containing plasmid MMR model was elaborated using a known protocol for incorporating oligonucleotides containing specific DNA lesions into a gapped DNA plasmid. We modified this approach to generate a double-gapped plasmid, which can be applied to introduce a G4 structure as well as various functional elements and/or site-specific DNA lesions using combinations of synthetic oligonucleotides that fill the double-gapped plasmid molecule.

Here we obtained a plasmid DNA containing a parallel G4 formed by the d(GGGT)_4_ sequence and a monomethylated ecMutH recognition site. To overcome the kinetic barrier of G4 formation in B-DNA, we stabilized the G4 structure in the DNA plasmid due to the absence of a sequence complementary to the G4-motif. To detect the G4 structure in our plasmid constructs, we used the Taq polymerase stop assay and DMS probing followed by Taq polymerase primer extention as sensitive tools. The G4-containing plasmid along with control ones (lacking looped area or with an unstructured d(GT)_8_ insert instead of a G4 motif) were used to answer the question of whether G4 formation interferes with the main function of MMR, i.e., DNA cleavage. It has been proven that the G4 structure stabilized in plasmid DNA is not a signal for the initiation of the methyl-directed mismatch repair characteristic of *E. coli* and does not activate the endonuclease function of the ngMutL protein, a component of the methyl-independent MMR from *N. gonorrhoeae*.

The described DNA plasmid models are more useful for studying multicomponent G4-driven biological processes than isolated G4 structures. Their advantage lies in the ability to (i) easily vary the number of functional elements, (ii) use different types of G4 structures and vary the distance between them and the selected functional moieties, and (iii) avoid nonspecific interactions of the repair proteins or other participants of processive cellular mechanisms with the ends of the DNA duplex.

## Figures and Tables

**Table 1:** Primary structures of the oligonucleotides used to construct DNA plasmids and primers for the Taq polymerase stalling assay.

Oligonucleotide	Sequence (5′-3′)
17G/T	pTCAGCACCTAGGGTGCC
17G/C	pTCAGCACCCAGGGTGCC
23GATC	pTGAGCCATATGCTCGAGGATCCC ^1^
42G4	pTGAGCCATATGTTGGGTGGGTGGGTGGGTTCTCGAGGATCCC ^1,2^
42loop	pTGAGCCATATGTTGTGTGTGTGTGTGTGTTCTCGAGGATCCC ^1,2^
Primer G	TAMRA-TATGCTAGTTATTGCTCAGCGG
Primer C	TAMRA-CTTTAAGAAGGAGATATACCATGGG

^1^ The unmethylated sequence d(GATC) of the ecMutH recognition site is underlined. ^2^ The G4 motif d(GGGT)_4_ and d(GT)_8_ sequence of the same size, which is unable to form a stable G4 structure, are shown in pink.

**Table 2:** Combinations of oligonucleotides inserted into double-gapped Dcm- and Dam-methylated pUC plasmid and names of resulting plasmids.

Name of the Plasmid	17-nt Gap	23-nt Gap
pUC-MMR	Unmodified Dcm- and Dam-methylated pUC-MMR plasmid	
G/T-pUC	17G/T	23GATC
G/C-pUC	17G/C	23GATC
G4-pUC	17G/C	42G4
loop-pUC	17G/C	42loop

## Data Availability

Not applicable.
